# Amyloidogenic biomarkers in Down syndrome

**DOI:** 10.1002/alz.089452

**Published:** 2025-01-09

**Authors:** Jessyka Maria de França Bram, Augusto Magno Tranquezi Cordeiro, Tamires Alves Sarno, Leda Leme Talib, Wagner Farid Gattaz, Orestes Vicente Forlenza

**Affiliations:** ^1^ Laboratory of Neurosciences, Institute of Psychiatry, Faculty of Medicine, University of São Paulo, Sao Paulo, Sao Paulo Brazil; ^2^ Instituto de Psiquiatria do Hospital das Clínicas da Faculdade de Medicina da Universidade de São Paulo, São Paulo, São Paulo Brazil; ^3^ Institute of Psychiatry, Faculty of Medicine, University of São Paulo., Sao Paulo, São Paulo Brazil; ^4^ Laboratory of Neurosciences (LIM27), Departamento e Instituto de Psiquiatria, Hospital das Clínicas, Faculdade de Medicina da Universidade de São Paulo, São Paulo Brazil; ^5^ University of São Paulo, São Paulo, Brazil, Sao Paulo, São Paulo Brazil; ^6^ Laboratory of Neuroscience (LIM27), Departamento e Instituto de Psiquiatria, Hospital das Clínicas, Faculdade de Medicina da Universidade de São Paulo, São Paulo, São Paulo Brazil

## Abstract

**Background:**

Due to the genetic characteristics of Down syndrome (DS), it is directly associated with a group of clinical manifestations resulting from premature aging and may present patterns of Alzheimer’s disease (AD). Thus, this study aimed to investigate AD biological markers in peripheral blood samples from adults and elderly individuals with DS and compare them with individuals with normal karyotype.

**Methods:**

The DS group was subclassified into 55 SD without evidence of cognitive decline (DSNC) and 27 DS with cognitive decline (SDAD). Two comparative groups composed of euploid individuals were constituted, 23 elderly with AD (AD) and 76 adults and elderly with normal cognition (Control). AD biomarkers were determined in platelets and plasma, including the Amyloid Precursor Protein ratio (APPr) between secreted 130‐ and 110kDA peptides, the protein expression of ADAM10, BACE1 and PSEN1, as well as levels of Aβ40, Aβ42 and between Aβ42/Aβ40 ratio.

**Results:**

Results demonstrated increased APPr for both DS groups compared to euploids. Lower expression of the APP130 and 110kDa fragments was observed in both DSNC and DSAD, and both fragments showed levels between 6 and 7 times lower in DSAD compared to DSNC. A reduction in the expression of ADAM10, BACE1 and PSEN1 was observed in individuals with DS compared to euploids, with no difference between DSNC and DSAD. AD subgroup had higher levels of BACE1 when compared to the Control. A higher plasma level of Aβ40 and a lower Aβ42/Aβ40 ratio were identified for both DSNC and DSAD groups, in relation to the Control. Using CART model, the combination of APP130kDa biomarkers and the Aβ42/Aβ40 ratio proved to be relevant for separating groups in terms of cognitive impairment, through the classification APP130kDa < 0.9, Aβ42/Aβ40 ≥ 0.409 and APP130 ≥ 0.77 or APP130kDa < 0.9, Aβ42/Aβ40 < 0.409, with a diagnostic accuracy of 79.9% (sensitivity: 30.6%; specificity: 98.5%; Kappa concordance coefficient: 0.365).

**Conclusion:**

The results of this study demonstrate that people with DS can show different patterns of expression of the proteins involved in the amyloid cascade, detectable even in the absence of cognitive decline, suggesting DS as a good predictive model of AD.

